# APFiLoc: An Infrastructure-Free Indoor Localization Method Fusing Smartphone Inertial Sensors, Landmarks and Map Information

**DOI:** 10.3390/s151027251

**Published:** 2015-10-26

**Authors:** Jianga Shang, Fuqiang Gu, Xuke Hu, Allison Kealy

**Affiliations:** 1 Faculty of Information Engineering, China University of Geosciences, Wuhan 430074, China; E-Mail: huxuke2012@163.com; 2 National Engineering Research Center for Geographic Information System, Wuhan 430074, China; 3 Department of Infrastructure Engineering, University of Melbourne, Melbourne, VIC 3010, Australia; E-Mails: fuqiangg@student.unimelb.edu.au (F.G.); a.kealy@unimelb.edu.au (A.K.)

**Keywords:** indoor localization, infrastructure-free, pedestrian dead reckoning, augmented particle filter, unsupervised clustering, landmark recognition

## Abstract

The utility and adoption of indoor localization applications have been limited due to the complex nature of the physical environment combined with an increasing requirement for more robust localization performance. Existing solutions to this problem are either too expensive or too dependent on infrastructure such as Wi-Fi access points. To address this problem, we propose APFiLoc—a low cost, smartphone-based framework for indoor localization. The key idea behind this framework is to obtain landmarks within the environment and to use the augmented particle filter to fuse them with measurements from smartphone sensors and map information. A clustering method based on distance constraints is developed to detect organic landmarks in an unsupervised way, and the least square support vector machine is used to classify seed landmarks. A series of real-world experiments were conducted in complex environments including multiple floors and the results show APFiLoc can achieve 80% accuracy (phone in the hand) and around 70% accuracy (phone in the pocket) of the error less than 2 m error without the assistance of infrastructure like Wi-Fi access points.

## 1. Introduction

Location is the most crucial context in mobile and ubiquitous computing [1], and how to obtain and infer the location is the key to location-aware applications. Indoor environments are main scenarios of people’s activity and it is estimated that about 87 percent of people’s time [2] are spent indoors. Nowadays location-based context-aware systems such as mobile social network [3], Internet of Things (IoT) [4] have no longer been restricted in outdoor environments and are extending to indoor spaces. Compared to outdoor environments, indoor space is more complicated in terms of layout, topology and space constraint, and presents some particular characteristics [5]. For example, indoor space is closed and has the constraint from indoor components. This leads to the problem that Global Navigation Satellite Systems (e.g., GPS, GLONASS, BEIDOU) cannot work indoors. Meanwhile, localization solutions exploiting mobile communication network can not meet the demand for high accuracy of indoor applications.

In recent decades, researchers have developed many indoor localization solutions [6,7,8,9,10,11], which differ with each other in terms of localization techniques used, coverage, accuracy, and cost of deployment and maintenance. Wi-Fi location fingerprinting is one of the most widely used indoor localization techniques since it can make use of existing Wi-Fi infrastructure and provide a relatively ideal accuracy. However, the need to collect fingerprints, which is labor-intensive and time-consuming, limits its applicability. Research on reducing the effort of collecting fingerprints has been done in [12,13] by modeling the constraints imposed by the physics of wireless propagation or by combining the signal characteristics with users’ movement.

Another popular technique for indoor localization is PDR (Pedestrian Dead Reckoning). Nowadays most smartphones integrate varying types of sensors such as accelerometer, magnetometer, gyroscope, and even barometer, making it possible for us to use these sensors to locate and track a person with a smartphone. Given the initial location, PDR can give the step stride length and heading of a user, which can be used to calculate his or her real-time location [14,15]. This is especially useful for locating and navigating in the blind areas of wireless signal. However, PDR needs to be calibrated periodically since its error increases over time. This can be directly done by the result of GPS [16] in outdoor environments and indirectly by the received signal strength of Wi-Fi [17], structures of buildings [18,19] in indoor environments.

Although a lot of research has been done in this field, some critical issues still need to be explored to enhance the accuracy and applicability. Most existing indoor localization solutions rely on Wi-Fi infrastructure, which are inapplicable in environments without Wi-Fi coverage or where no enough Wi-Fi access points (APs) are available. Although Wi-Fi infrastructure is becoming widespread, it still true that there are many areas without Wi-Fi covered because of cost and safety related concerns. Also, Wi-Fi is regarded as an energy-hungry technology, which would reduce the life of a smartphone’s battery. In addition, existing research work focuses mainly on either utilizing the constraint of a floor plan or landmarks to enhance the location accuracy. In principle, the floor plan and landmarks can complement each other. While the floor plan gives coarse information, *i.e.*, the boundary between rooms, it does not provide information about the interior structures within a room. Landmarks can reflect indoor constraints imposed by obstacles. We believe that using both the floor plan and landmarks can significantly improve location accuracy.

To take advantage of the characteristics of complex indoor space and eliminate the reliance on Wi-Fi infrastructure, we develop an indoor localization solution called APFiLoc, which uses an augmented particle filter to integrate readings of inertial sensors, map information, and landmarks. The key idea behind this study is to use both map information and landmarks to eliminate invalid particles, *i.e.*, those passing through walls or other barriers, and to amend the accumulated error of PDR, so as to improve the location accuracy. In summary, our main contributions are as follows:

- We propose APFiLoc—a low cost, smartphone-based framework for indoor localization. APFiLoc has no need for Wi-Fi infrastructure and is accomplished using an augmented particle filter. It fuses not only PDR and map information, but also landmarks. Such a fusion makes it possible to adapt the step stride length of different users and to be independent of smartphones’ attitudes. Different from most existing projects, which only consider single-floor environments, APFiLoc can work in complex environments including multiple floors.
- We propose a clustering method depending on distance constraints, which can generate magnetic landmarks and directional landmarks in an unsupervised way (see Section 5 for details). Compared to traditional K-means clustering, our method does not need the knowledge of the number of initial clusters and can iteratively produce clusters needed under the constraint.
- We evaluate the influences of landmarks and map information on its performance by a series of real-world experiments, which were conducted in a typical office building consisting of multiple floors. Experimental results show that APFiLoc could achieve 80% accuracy (phone in the hand) and around 70% accuracy (phone in the pocket) of the error less than 2 m error without the assistance of Wi-Fi APs.

This study postulates that a floor plan describing experimental areas is available, which we think does not need extra efforts in many cases since indoor maps are basic information to support location-based services. The employment of a map enables us to constrain the routes of particles and to obtain the positions of seed landmarks. It is assumed that the initial deviation between user heading and phone heading is known. This assumption may not be trivial as it requires the user to do some initial calibration and if not done correctly will affect future performance.

The rest of this paper is structured as follows: Section 2 reviews research work regarding sensor fusion. Section 3 presents the architecture of APFiLoc and its three key components. The subsequent three sections expand on each of these components, beginning with PDR (corresponding to Motion Estimator in APFiLoc), followed by Landmark Detection and Augmented Particle Filter. Section 7 presents experimental results for evaluating the proposed solution. Finally Section 8 concludes the paper and provides directions for future work.

## 2. Related Work

This paper intersects with a number of past research projects especially those on the indoor positioning. Here we mainly focus on calibration-free positioning techniques and Bayesian filtering -based sensor fusion approaches.

### 2.1. Calibration-Free Positioning

Wi-Fi location fingerprinting is one of the widely-used methods for indoor positioning since it can make use of existing Wi-Fi access points. However, collecting fingerprints is a labor-intensive and time-consuming task, which is inapplicable for some applications. EZ [12] is one of the pioneers to attempt indoor positioning without explicit pre-deployment effort. It assumes that the smartphone can receive occasionally the GPS signal and report a location fix. These observations together with the Wi-Fi signal strength received by the smartphone at different (unknown) locations are constrained by the physics of wireless propagation. Then the user’s location can be determined by modeling these constraints and using a genetic algorithm to solve them. While EZ reports a median positioning error between 2 m and 7 m, which are not accurate enough to distinguish various rooms. Moreover, the fact that Wi-Fi signal is susceptible to obstacles or other signals leads to the constraint on its widespread use.

WILL [13] exploits both RF signal characteristics and user motions to build up a radio floor plan which is previously generated by site survey. The core idea behind WILL is using user motions to connect independent radio signature under certain semantics. The position of a user can be obtained by matching the logical floor plan and the real floor plan. It reports a room-level accuracy, but such an accuracy cannot meet the demand of indoor applications such as navigation in narrow spaces. UnLoc [20] makes use of signatures inherent in an indoor environment, the locations of these signatures are called landmarks which we adopted in the paper. These landmarks are used to calibrate the accumulated error of PDR, which in turn improve the locations of landmarks. By fusing PDR, urban sensing and Wi-Fi-based partitioning, UnLoc bypasses the need for war-driving and achieves less than 2 m error. However, it may suffer from inaccurate positioning when two landmarks are far away from each other. Besides, since magnetic and inertial sensor landmarks in UnLoc are dependent on Wi-Fi partition, it fails to work in an environment without Wi-Fi coverage. Zee [21] leverages the inertial sensors built in a smartphone to locate the user while simultaneously collecting Wi-Fi fingerprints. The intuition behind Zee is collecting training data without any explicit effort and employing the augmented particle filter to infer location. Yet Zee also relies on the Wi-Fi infrastructure and cannot function normally within areas that are not covered by Wi-Fi APs.

In APFiLoc, we bypass labor-intensive calibration by utilizing landmarks that are derived from inertial sensors, user motions and map information. In contrast to past studies, which commonly depended on Wi-Fi infrastructure, our solution can achieve less than 2 m error in our test scenarios without the assistance of Wi-Fi. This is attributed to the utilization of both map information and landmarks.

### 2.2. Sensor Fusion Based on Bayesian Filters

Bayesian filtering approaches are usually used to integrate multiple sensors or measurements to achieve higher localization accuracy, mainly including Kalman filters and particle filters. The Kalman filter was used to fuse Wi-Fi, PDR, and landmarks in [22]. Wi-Fi fingerprinting method was used to provide initial location and exact location while PDR was utilized to offer relative location. By combining these two methods, the authors achieved an average localization accuracy of 1 meter in their testbeds. A hybrid structure that consists of a Kalman filter and a particle filter was developed to combine PDR and Wi-Fi signal strength measurements [23]. The Kalman filter was utilized to provide real-time position and infer position when a user is in the areas without Wi-Fi coverage, while the particle filter was used to correct the drift on the inertial sensors. The typical WLAN-based indoor positioning systems was extended via using the particle filter to integrate an MEMS accelerometer and map information [24]. The authors of [25] demonstrated a particle filter-based end-to-end localization system, which is infrastructure free, phone position independent, and easy to deploy. By using the proposed personalized step model and heading inference method, they achieved a mean accuracy of 1.5 m for the in-hand case and 2 m for the in-pocket case in a single-floor annular area.

In this paper, we use the particle filter to integrate different kinds of information in order to eliminate the reliance on Wi-Fi infrastructure. Both landmarks and map constraints are considered in this study. In general, maps provide room-level information in a building, which are commonly about walls and corridors, rather than finer information about other indoor obstacles. Compared with maps, landmarks can to some extent reflect more fine-grained indoor structures such as doors, corners, and spots of metal equipments, which means that incorporating them can achieve better localization accuracy. Besides, by adding the stride model parameter and heading into the state vector of the particle filter, our solution can adapt different users’ stride and be independent of smartphones’ attitudes.

## 3. Architecture of APFiLoc

**Figure 1:**
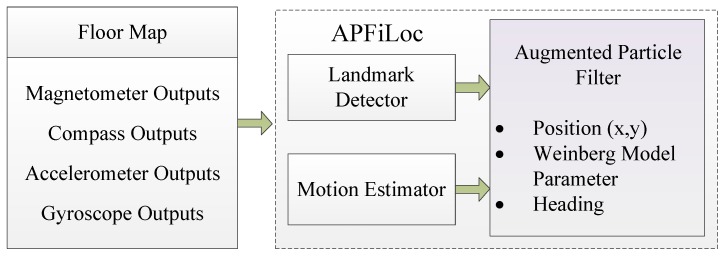
Architecture of APFiLoc.

We begin with a top level overview of APFiLoc, focusing mainly on three core components: Motion Estimator, Landmark Detector, and Augmented Particle Filter (APF), as shown in the Figure 1. Specifically, Motion Estimator uses accelerometer, compass, and gyroscope data to compute user stride length and heading, which are fed to the augmented particle filter for fusion.

Landmark Detector is responsible for generating landmarks that are used to correct the accumulated error and adjust Weinberg model parameter. There are two core algorithms in Landmark Detector: the least square support vector machine (LS-SVM) and the clustering algorithm based on distance constraints. LS-SVM is used to classify seed landmarks and a clustering method is developed for recognizing organic landmarks, which will be elaborated in Section 5.

In order to enable APFiLoc to adapt to different users’ step characteristics and to be independent of smartphones’ attitudes , the particle filter is extended by adding Weinberg model parameter and heading to the state vector, which we call APF. The key function of the APF is to integrate outputs from above two components and floor map. With such information, the APF can estimate real-time positions and heading, and adjust Weinberg model parameter, which in turn can refine the positions of organic landmarks. The positions of organic landmarks may not be precise at the beginning, but they can constantly converge over time. The details about the APF will be further discussed in Section 6.

## 4. Motion Estimation

Usually, using the repetitive and periodical characteristics of users’ walking can obtain relatively accurate stride length estimation [21], but we find that the stride length varies from person to person. To tackle this problem, the APF is utilized to adaptively adjust the parameter of stride length model. Another issue of PDR is heading deviation. We consider two common poses of a user carrying his or her phone in this paper: putting in the pocket and holding in the hand. When the user holds his phone in the hand, his walking direction is considered to be consistent with the *Y*-axis of the smartphone. In this case, we use Kalman filter to fuse compass readings and gyroscope readings to eliminate the effect of ferromagnetic materials on compass readings and the drift of the gyroscope. If users put their phone in the pocket, the heading inference method presented in [25] is adopted. Next, we elaborate the algorithms involved in the Motion Estimator, including step detection based on repetition characteristics, stride length calculation and heading estimation.

### 4.1. Step Detection

The smartphone we used in the study includes a 3-axis accelerometer at 16 HZ with *x* and *y* axis being parallel to the width and length of the smartphone’s screen respectively. To enable the step detection method not to be influenced by the smartphone’s orientation, we only utilize the magnitude of the acceleration:

(1) $$a_{t}=\sqrt{{a_{x_{t}}}^{2}+{a_{y_{t}}}^{2}+{a_{z_{t}}}^{2}}$$

where $a_{x_{t}},a_{y_{t}},a_{z_{t}}$ are the accelerometer readings along the *X*-axis, *Y*-axis and *Z*-axis at time *t*, respectively. A simple high pass filter and a low pass filter [26] are applied to the magnitude of the acceleration to remove the Earth’s gravity and the noise of the signal, respectively. Traditional step detection methods, including peak detection and zero crossings [27], need to adjust the parameter according to the smartphone’s placement, orientation, and walking characteristics of the user, which may result in large error in practice [26]. In order to enable the step detection algorithm be independent of the smartphone’s placement, *i.e.*, whether the user is carrying the smartphone in the pocket, hand, *etc.*, we utilize repetitive nature of walks to judge whether a step event happens, more details about this are available in [21].

### 4.2. Stride Length Calculation

In general, stride length varies from person to person due mainly to different walking characteristics. This makes it difficult to use the same model parameters to precisely estimate users’ stride length. Although there are Weinberg model, Kim model and step counting methods available to compute the stride length, we have to adjust the model parameter to adapt various users’ walking characteristics. Therefore, we design a self-adaptive stride length model to tackle this problem. The Weinberg model parameter is added to the state vector of APF, and there is no need to manually modify the parameter to obtain accurate stride length for different users. Such an improvement makes it possible to take full advantage of landmarks and map information to update the parameter of stride length model. The basic Weinberg model [28] is described below:

(2) $$s_{k}=\alpha \cdot \sqrt[{4}]{a_{max}-a_{min}}$$

where *α* is a constant and $a_{max}$, $a_{min}$ are the maximum acceleration and the minimum acceleration for each step, respectively. Our approach takes *α* as the initial value and then iteratively adjusts it in the light of map information and landmarks that are encountered by the user.

### 4.3. Heading Estimation

The smartphone’s compass can provide the angle of its orientation relative to the perceived north. When the smartphone is put in the pocket, the method in [25] is adopted to infer the user’s walking direction. If the user holds the phone in the hand, we utilize the Kalman filter to fuze both compass data and gyroscope data. The combination of the compass and gyroscope can not only make up their respective limitation but also improve the accuracy of heading computation. This combination allows us to obtain a more accurate heading estimation since it eliminates the effect of metals on compass readings and the drift of the gyroscope [29]. The key processes are summarized as follows.

**Prediction**:

(3) $$\theta_{k}^{-}=\theta_{k-1}^{-}-\dot{\theta_{k}}\cdot \Delta T$$

(4) $$P_{k}^{-}=P_{k-1}+Q$$

**Update**:

(5) $$\theta_{k}=\theta_{k}^{-}+K_{k}\cdot \left(\theta_{k}^{{}^{\prime }}-\theta_{k}^{-}\right)$$

(6) $$K_{k}^{-}=P_{k}^{-}/\left(P_{k}^{-}+R\right)$$

(7) $$P_{k}=\left(I-K_{k}\right)\cdot P_{k}^{-}$$

where $\dot{\theta_{k}}$ and $\theta_{k}$ indicate the gyroscope reading and heading computed at the $k_{th}$ step, respectively. ΔT is the sampling interval of the gyroscope, $\theta_{k}^{{}^{\prime }}$ is the angle from the compass. *Q* and *R* are the covariance of process and measurement noise, respectively. $K_{k}$ represents the Kalman gain and $P_{k}$ is the error covariance matrix.

## 5. Landmark Detection

This section presents the details of detecting landmarks. There are two kinds of landmarks in this paper: seed landmarks and organic landmarks, similar to those in [20]. Both seed landmarks and organic landmarks are location points in the environments where users are forced to behave in a distinct motion state or some types of sensor readings present a predictable and distinguishable pattern. The locations of seed landmarks (e.g., stairs, elevators, doors) are known or can be inferred from the building’s floor plan; while the locations of organic landmarks are initially unknown and will be inferred from users’ trajectories in a crowd-sourcing way. We design a clustering method based on distance constraints to learn organic landmarks in an unsupervised way, while we use the least square support vector machine (LS-SVM) to classify seed landmarks, which has been proven as the more effective supervised classification method [30] compared to Decision Tree, Bayesian Network using the Gaussian Mixture Model, Linear Discriminant Analysis.

### 5.1. Seed Landmarks

Since each kind of seed landmarks forces the user to behave in a distinct motion state, we can distinguish the type of seed landmarks according to users’ motion state. We define 6 motion patterns as shown in Table 1. The former two states (M1, M2) correspond to elevator seed landmarks, while M3 and M4 are relative to stair seed landmarks. The remaining two states (M5, M6) can help us set proper parameters to differentiate the former four states. When we recognize a user being in M1, we can immediately know his position by matching his state with these states that are related to seed landmarks.

**Table 1:** Motion state definition.

Motion State	Definition
M1	Going up elevators
M2	Going down elevators
M3	Going up stairs
M4	Going down stairs
M5	Walking
M6	Stationary

We adopt LS-SVM depended on RBF (radial basis function) kernel function [31] to classify the 6 states defined in Table 1. LS-SVM is a semi-supervised classification algorithm relied on kernel functions and has a relatively high recognition rate. Compared to general SVM, LS-SVM introduces a least square loss function and works with equalities to address linear systems rather than solving convex optimization problems. Here we provide a brief introduction of LS-SVM, and further details regarding LS-SVM can be found in [30].

The classification problem using LS-SVM is equal to solve the following optimization problem:

(8) $$min_{w,e,b}J\left(w,b,e\right)=\frac{1}{2}w^{T}w+\frac{1}{2}\gamma \sum_{i=1}^{N}e_{i}^{2}$$

subject to:

(9) $$y_{i}\left(w^{T}\varphi \left(x_{i}\right)+b\right)\geq 1-e_{i},i=1,\cdots ,N$$

where *J* is the least squares loss function, *w* is the weight vector, *γ* is a positive regularization parameter, *e* is a vector of error variables, φ(·) is a mapping function, and *b* is a bias term. The corresponding Lagrangian function for this problem is:

(10) $$L\left(w,b,e;\alpha \right)=J\left(w,b,e\right)-\sum_{i=1}^{N}a_{i}\left(y_{i}\left[w^{T}\varphi \left(x_{i}\right)+b\right]-1+e_{i}\right)$$

where *α* is a vector of Lagrange multipliers (or support values). By taking the conditions for optimality, we can finally obtain the LS-SVM classifier:

(11) $$y\left(x\right)=sign\left(\sum_{i=1}^{N}\alpha_{i}y_{i}K\left(x,x_{i}\right)+b\right)$$

where *K* is a positive definite kernel matrix. By transforming the original data into a higher dimensional feature space, different motion states can be classified.

### 5.2. Organic Landmarks

In order to recognize these organic landmarks without any explicit user effort, we adopt the idea of crowdsourcing that sensor readings of the smartphone are uploaded simultaneously when the user is moving. After pre-processing to these readings, features are extracted from sensor readings and then transferred together with corresponding positions from the APF into the clustering method we designed.

There are two types of organic landmarks in this paper: magnetic landmarks and directional landmarks. The former refers to those points where the magnetometer presents an outlier due to the effect of ferromagnetic materials. We define the location point where the average value of a window of magnetometer readings exceeds a threshold as a potential magnetic landmark. When detecting a potential magnetic landmark, we record the magnetometer readings and corresponding position from PDR at this point. The directional landmarks are detected by using both compass readings and gyroscope readings. Only when both the variation in compass readings over two neighboring moving windows and that in gyroscope readings over a moving window exceed an angle threshold and an angular threshold, respectively, we think this location point is a potential directional landmark and record the sensor readings and corresponding position at this point.

To learn these organic landmarks in an unsupervised way, we design a clustering algorithm depended on distance constraint. Traditional clustering methods [32] need the knowledge of the number of clusters, which is impractical in some cases where the number can not be determined in advance. In this paper, we attempt to replace this constraint with the distance constraint. The clustering algorithm is described in the following.

Let $Y=\{y_{1},y_{2},\cdots ,y_{n}\}$ represent unlabeled data (which are potential organic landmarks) to be classified, the corresponding distance constraint is $d(y_{i},y_{j})<r$ which indicates that only when the distance between two points is less than *r* , they can be clustered into the same cluster. Let $C_{1},C_{2},\cdots ,C_{k}$ indicate different clusters and we have no knowledge of the number of clusters. The pseudocode (Algorithm 1) describes the clustering algorithm.

**Algorithm 1** Organic Landmark Clustering.**Input:** a series of location points with the feature *d*_1_ or *d*_1_ exceeding the pre-set thresholds**Output:** a set of organic landmarks *C*
1:
**Initialization:** $C_{1}\leftarrow \left\{y_{1}\right\},k\leftarrow 1$
2:**for** *i* = 2 to *n* **do**3:    $j\leftarrow \min d\left(y_{i},center\left(C_{m}\right)\right)$4:    **if** $d\left(y_{i},center\left(C_{j}\right)\right)<r$ **then**5:        put $y_{i}$ into $C_{j}$,then update the center of $C_{j}$6:        **if** $\forall y^{\prime }\left(y^{\prime }\in C_{j}\right),d\left(y^{\prime },center\left(C_{j}\right)\right)>r$ **then**7:           put $y_{i}$ into a new cluster $C_{k=k+1}$8:        **end if**9:    **else**10:        put $y_{i}$ into a new cluster $C_{k=k+1}$11:    **end if**12:
**end for**
13:
**Adjustment:**
14:**repeat**:15:    **for** *i*=1 to *k* **do**16:        **for** j=i+1 to *k* **do**17:           **if** $d(center\left(C_{i}\right),center\left(C_{j}\right))<2r$ **then**18:               **if** $\exists \dot{y}\in C_{j},d\left(\dot{y},center\left(C_{i}\right)\right)<d\left(\dot{y},center\left(C_{j}\right)\right)$ **then**19:                   delete $\dot{y}$ from $C_{j})$ and update the centers of $C_{i}$, $C_{j}$20:               **end if**21:           **end if**22:        **end for**23:    **end for**24:    **if** $isEmpty(C_{i})$, *i* =1 to *k* **then**25:        delete $C_{i}$26:    **end if**27:**until** all samples are clustered into the clusters nearest to them


There are two core operations in the algorithm: Firstly, all the unlabeled data are classified into the cluster nearest to their positions under the distance constraint or into a new cluster when the constraint is not met. Then for all the clusters, iteratively adjust those elements which are closer to the center of the other clusters than they are to their own center and recalculate all the centers. After this, repeat the previous steps until all the data are distributed in a way that every data point in any given cluster is closer to its own center than it is to the center of any other cluster. To obtain the precise positions of organic landmarks, APFiLoc adopts the backtracking technique [33]. Every particle remembers its state trajectory, which is used to refine locations of organic landmarks. This can in turn improve subsequent location accuracy. Since the error computed by the APF is random, the location estimation of organic landmarks can converge with more trajectories available. To avoid the influence of fake organic landmarks, *i.e.*, when a user changes his heading on a straight corridor, we only consider those clusters whose quantity of elements is greater than a threshold as organic landmarks.

## 6. Augmented Particle Filter

In this section, we elaborate the APF used to fuse motion estimation, landmarks, and map information. The backtracking technique [33] is utilized in the APF to refine locations of organic landmarks, which can in turn improve subsequent location accuracy. When the particle $x_{k}^{i}$ at the $k_{th}$ step crosses walls or other obstacles, the previous state estimates back to $x_{k-m}^{i}$ can be improved via eliminating the invalid particle trajectories. Meanwhile, we extend the state vector of the APF by adding the parameter of Weinberg model and heading. The purpose of this is to adapt various step characteristics and to enable APFiLoc to be independent of smartphones’ attitudes.

To develop the details of the algorithm, let $X_{i}={\left(x_{i}\;y_{i}\;\alpha_{i}\;\theta_{i}\right)}^{T},i=1,2,\cdots ,N$ denote the state vector, where (x,y) is the coordinate and *α* is the parameter of Weinberg model. $\theta_{i}$ is the heading, which can be initially computed by using the method in [25]. The measurement model and state model can be written as:

**Measurement model**: $Z_{k}=\left(a_{k}\;\theta_{k}^{{}^{\prime }}\;\dot{\theta_{k}}\right)$ ,where $a_{k}$ is the acceleration at the $k_{th}$ step, $\theta_{k}^{{}^{\prime }}$ is the angle measured by the compass and $\dot{\theta_{k}}$ is the angular velocity measured by the gyroscope.

**State model**: $x_{k}^{i}=x_{k-1}^{i}+s_{k-1}^{i}\cdot \sin\left(\theta_{k}^{i}+\gamma_{i}\right)$, $y_{k}^{i}=y_{k-1}^{i}+s_{k-1}^{i}\cdot \cos\left(\theta_{k}^{i}+\gamma_{i}\right)$, where $s_{k-1}^{i}$ is the stride length computed by the Weinberg model with the parameter $\alpha_{i}$, $\alpha_{i}∼N\left(\alpha_{0},\sigma_{\alpha }^{2}\right)$. $\sigma_{\alpha }$ is the standard deviation of the Weinberg model parameter and $\gamma_{i}$ is a zero-mean Gaussian noise. $\theta_{k}^{i}$ is the output of the heading inference method in [25] (phone in the pocket) or the output of the Kalman filter (phone in the hand). The key steps of the APF are presented below.

(1) **Initialization**. Draw *N* samples $x_{0}^{i}\;\left(i=1,2,\cdots ,N\right)$ according to the initial posterior probability density function and assign the initial weight for each particle using $w_{0}^{i}=1/N$.

(2) **Prediction**. Compute the state $x_{k}^{i}$ at the $k_{th}$ step for each particle based on the state model.

(3) **Weight calculation**. In general, there are two scenarios that we need to recalculate the weight. First, the weight of a particle will be assigned to zero when it crosses walls or obstacles. The second is that when encountering a landmark, the weights of particles will be recalculated according to the distances from this landmark. The nearer a particle to this landmark, the greater its weight. The basic equation for using landmarks to recalculate weights is defined as:

(12) $$w_{k}^{i}=\left\{\begin{array}{cc} 0, & \text{if}\;\text{crossing}\;\text{a}\;\text{wall}\;\text{or}\;\text{obstacle}\\ w_{k-1}^{i}\cdot \frac{1}{\sqrt{2\pi }\sigma }e^{-\frac{{∥X_{z_{k}}-X_{z_{k}^{{}^{\prime }}}∥}^{2}}{2\sigma^{2}}}, & \text{if}\;\text{encountering}\;\text{a}\;\text{landmark} \end{array}\right.$$

where $X_{z_{k}}$ is the coordinate of the landmark. $X_{z_{k}^{{}^{\prime }}}$ is the estimated coordinate using new measurements and *σ* is the corresponding standard deviation. After this, the weights need to be normalized by using the equation below.

(13) $$\dot{w_{k}^{i}}=w_{k}^{i}/\sum_{j=1}^{N}w_{k}^{j}$$

(4) **State estimate**. The state can be obtained via the following equation.

(14) $$X_{k}^{{}^{\prime }}=\sum_{i=1}^{N}\dot{w_{k}^{i}}\cdot x_{k}^{i}$$

(5) **Resampling**. The basic idea of resampling is to replace particles of small weights with those of large weights. It involves producing a new set of particle when the degeneracy problem arises, while $\alpha_{i}$ is not updated at this stage.

(6) **Backtracking**. Each particle has to remember its trajectory in the localization process. When a particle $x_{k}^{i}$ is detected being invalid at the $k_{th}$ step, its previous state estimates back to $x_{k-m}^{i}$ can be refined, thereby improving the locations of organic landmarks.

## 7. Evaluation

**Figure 2:**
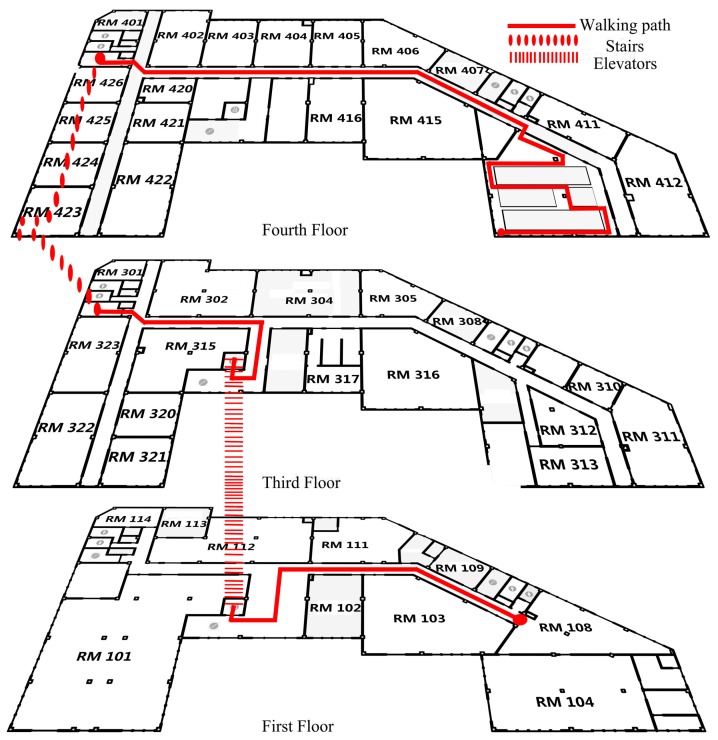
Ground truth paths in experiments.

The proposed APFiLoc solution was evaluated by a series of experiments conducted in an office building, occupied by the China National Engineering Research Center for Geographic Information System. Figure 2 shows the experimental scenarios, in which the ground truth paths are marked in solid lines (horizontal motions) and dotted lines (vertical motions). This office building consists of four floors with an area of 3300 square meters for each floor, which is a typical office environment, including an elevator, staircases, corridors, office rooms and electronic equipment. The length of pre-set test path is about 290 m, going through 3 floors of this building selected.

The device we used is the Samsung Galaxy S III phone equipped with accelerometer, magnetometer, gyroscope, and barometer. Two testers of different heights were asked to walk along this pre-set paths for 10 times with the phone in the hand and in the pocket, respectively. During the experiments, the testers were required to report the pre-set markers they encountered to evaluate the location accuracy. The detailed location accuracy evaluation method will be further discussed in Section 7.2.

### 7.1. Classification and Recognition of Landmarks

#### 7.1.1. Seed Landmarks

To classify and recognize seed landmarks, we use the accelerometer readings $a_{x},a_{y},a_{z}$ and the barometer reading *b* to deduce features, including the total acceleration *a*, horizontal acceleration $a_{h}$, vertical acceleration $a_{v}$, the variation of the barometer reading $d_{b}$ and their respective mean and covariance. These features are extracted over a moving window, which are then fed to the LS-SVM. If a user is detected in a motion pattern related to seed landmarks, e.g., upstairs or downstairs, we would compare the location estimated from PDR and seed landmarks’ locations derived from the map, and select the location of the nearest seed landmark to calibrate the estimated location.

**Figure 3:**
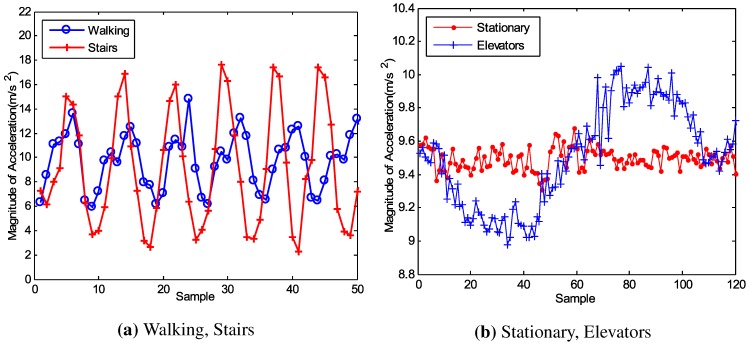
Acceleration of different state.

**Figure 4:**
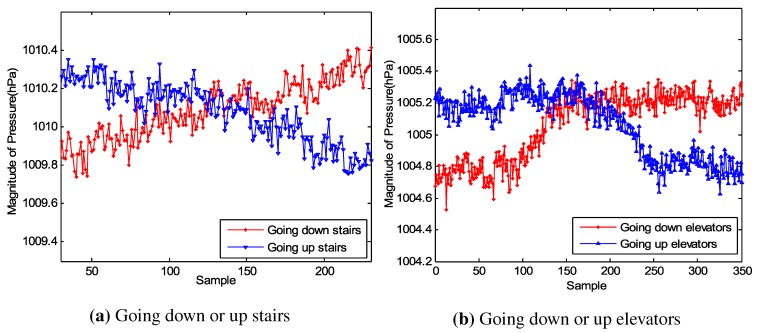
The change on the barometer when a user is moving from one floor to another.

Figure 3a,b indicate that the acceleration varies from state to state and it is easy to distinguish stairs and walking, elevators and stationary. However, we observe that it is difficult to tell the difference between M1 and M2 (or M3 and M4) simply through the acceleration. The emergence of the barometer that has been built in many smartphones provides us with the opportunity to address this question. Figure 4a,b give the changes on the barometer when the user is moving from one floor to another by stairs and elevators, respectively. The barometer reading is affected by weather, temperature and humidity, which means that this reading cannot be used to judge on an absolute scale which floor a user is lying. However, the variation between two floors in a building is constant, which stays at about 0.5 hPa for our test building. This relative variation is reliable to distinguish different floors.

The 5-fold cross validation [34] was used to assess the performance of LS-SVM adopted by APFiLoc. Experimental data was collected by a Samsung Galaxy S III smartphone. After pre-processing these data, the feature values are computed over a moving window. There are 1379 samples in total. These samples are randomly partitioned into 5 equal size subsamples. The cross validation process is repeated 5 times, with each of 5 subsamples used once as the validation data for testing the model while the remaining 4 subsamples as training data. The estimate can be obtained by averaging the 5 results from the folds. We adopt the toolbox LS-SVMlab [35] to analyze the performance of LS-SVM. By experimental analysis, we finally select $\left\{\mu_{a},\sigma_{a}^{2},\mu_{a_{v}},d_{b}\right\}$, namely mean and variance of the total acceleration, mean of vertical acceleration, difference in the barometer readings, as the features for classification, achieving a recognition rate of 97.5%, which is good enough for detecting seed landmarks.

#### 7.1.2. Organic Landmarks

**Figure 5:**
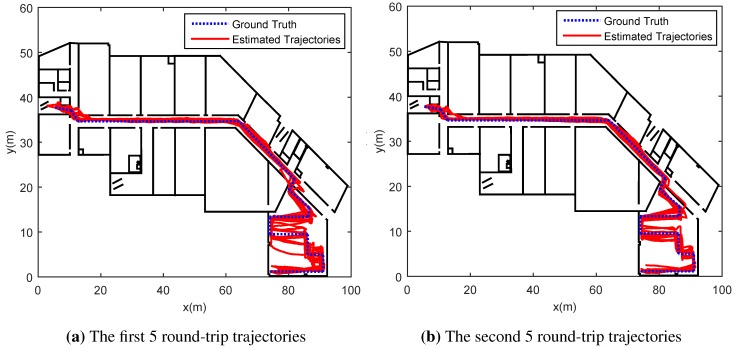
The trajectories in the fourth floor.

In Section 5.2, we introduced magnetic landmarks and directional landmarks. The threshold for magnetic landmarks is set to 65 uT while the thresholds for directional landmarks are set to $30^{\circ }$ (for compass readings) and 0.5 rad/s (for gyroscope readings). The 10 collected traces are divided into two groups. Each group includes 5 traces, as shown in Figure 5a,b, in which the blue dotted line indicates the ground truth while the red lines are trajectories estimated. Figure 5a shows that the trajectories of first 5 round-trip traces, while Figure 5b illustrates trajectories for the second 5 round trip traces. Initially, there is no knowledge of organic landmarks, thereby the trajectories estimated deviate significantly from the ground truth. The subsequent trajectories are computed using the knowledge of organic landmarks discovered by former traces. Therefore, the deviation between the ground truth and trajectories estimated reduces with more traces available. The results shown in Figure 5b outperform those in Figure 5a.

**Figure 6:**
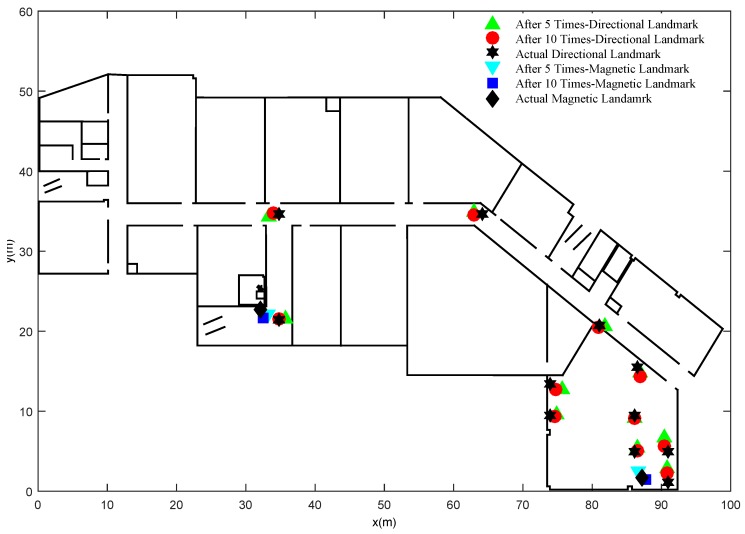
Organic landmarks recognized by the proposed clustering algorithm.

Figure 6 gives the information about organic landmarks’ actual locations, locations estimated from 5 traces and that from 10 traces. Overall, the locations estimated from 10 traces are closer to actual locations than that from 5 traces. After 10 traces, all the organic landmarks, including magnetic landmarks and directional landmarks, are recognized by the proposed algorithm without the knowledge of the number of clusters.

We also found out that magnetic landmarks are sensitive to the threshold. To eliminate the false magnetic landmarks, the threshold is set to 65 uT after experimental analysis. Besides, there are lots of factors that influence the detection of magnetic landmarks. In most cases, magnetic landmarks can be detected only if the device is close to ferromagnetic materials (usually less than 50 cm). For directional landmarks, the detection error often arises from users’ arbitrary turning. For instance, the user turns around at the corridor where there are actually no turns or corners. To tackle such error, we only use the potential organic landmarks that are encountered by the user for more than 5 times to compute the exact location of an organic landmark.

### 7.2. Localization Results

**Figure 7:**
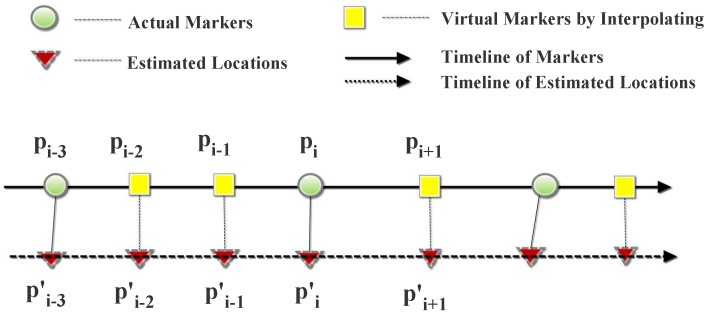
The accuracy evaluation method.

In this section, we assess the localization accuracy of APFiLoc and analyze the effect of landmarks and map information on the performance. The number of particles for APFiLoc *N* is set to 1000. The initial value and standard deviation of Weinberg model parameter *α* are set to 0.66 and 0.07, respectively. APFiLoc interacts with the user to obtain the initial location. To precisely assess the location accuracy, we used the interpolation method to obtain the actual locations between two makers according to sampling interval and timestamps, as shown in Figure 7. The distance between two markers was 2 meter in this study. After computing the localization error at each location point, the overall error can be calculated using the following formula:

(15) $$e=\sum_{i=1}^{N}e_{i}=\sum_{i=1}^{N}∥L\left(p_{i}\right)-L\left(p_{i}^{{}^{\prime }}\right)∥$$

where $L(p_{i})$ is the location of the $i_{th}$ marker (including the virtual markers generated by interpolating), and $L(p_{i}^{{}^{\prime }})$ is the estimated location corresponding to the $i_{th}$ marker.

**Figure 8:**
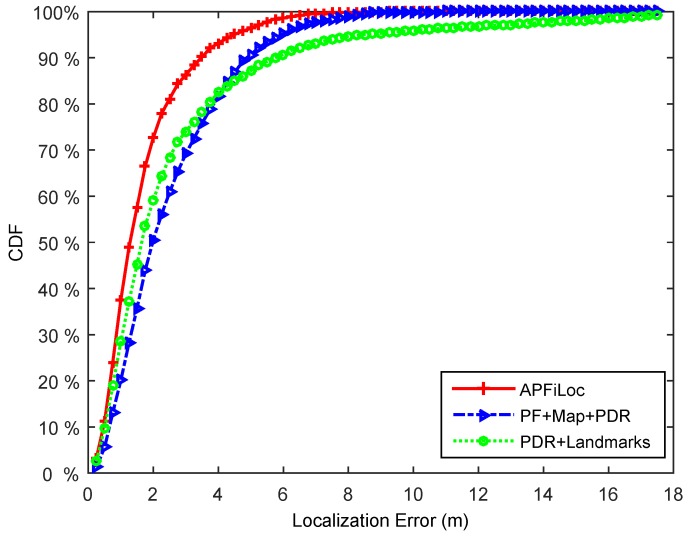
Performance comparison of different methods (Phone in the hand).

**Figure 9:**
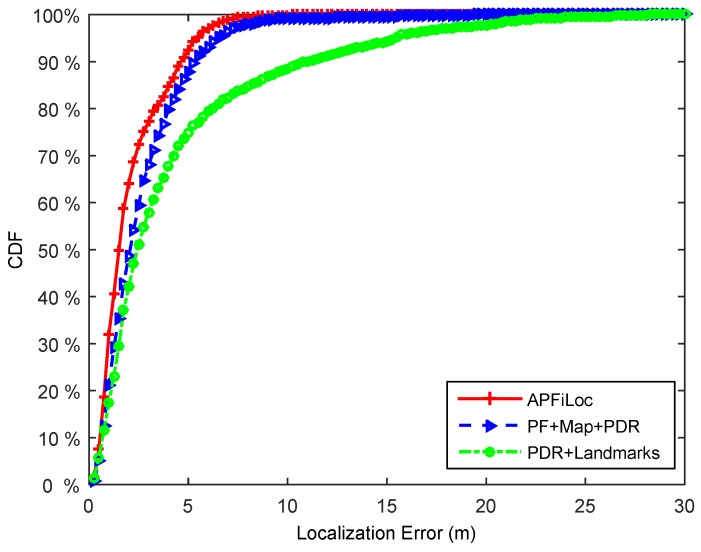
Performance comparison of different methods (Phone in the pocket).

We compare the performance of APFiLoc with PDR + Landmarks and Particle Filter + Map Information + PDR solutions. Figure 8 and Figure 9 illustrate the localization results of different solutions in the case of the phone in the hand and that in the pocket, respectively. It shows that in our test environment, APFiLoc outperforms other two solutions in both cases. Specifically, in the case of the phone in the hand, APFiLoc (red line with plus sign) can achieve 80% accuracy with the error less than 2 m compared to 68% (blue line with triangle sign) for PF + Map + PDR and 60% (green line with dot sign) for PF + Landmarks. The figure for the solution PF + Map + PDR is slightly less than that for PDR + Landmarks until it reaches 82% with the error less than 4 m, when the figures for both solutions are equal and after that the accuracy for PF + Map + PDR is higher than that for PDR + Landmarks. When the phone is put in the pocket, the overall accuracy for each solution decreases by 12% since the heading estimation is less accurate compared to the case that the phone is in the hand. In the case, APFiLoc still performs best in terms of accuracy, followed by PF + Map + PDR and then by PDR + Landmarks.

From the Figure 8 and Figure 9, we can also see that both map information and landmarks are useful in improving localization accuracy. Map information can enhance the accuracy by imposing constraint on the possible paths and eliminating invalid particles. While landmarks contribute to the reduction of location error, which is especially obvious when users lie in a large room. For the corridor environments, landmarks are less useful since they are naturally included in the map information. However, most maps only have the coarse information, which is typically boundaries of rooms. For a larger office room (e.g., the area is more than 100 square meters), only map information is not enough to provide a good localization result. In this case, landmarks, which can reflect the interior structures of a room, are necessary for improving localization accuracy. Overall, map information and landmarks can complement each other, and combining both can accomplish a better localization result.

**Figure 10:**
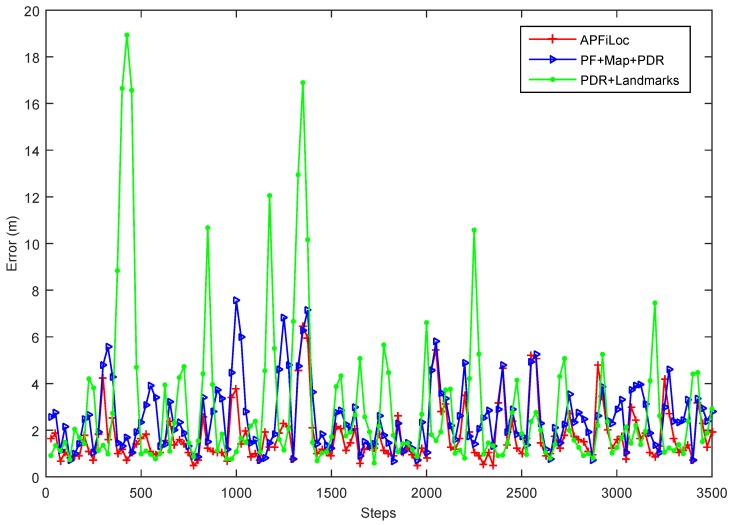
The errors of different methods over the distance moved.

The accuracy increases with more landmarks being recognized until all landmarks are successfully detected and recognized. Take the case that the phone was held in the hand as an example, the accuracy change of different localization methods over time was shown in Figure 10. The data for Figure 10 is the accumulated distances or steps conducted by one participant moving along the pre-set experimental paths for 5 times. To be visually clear, we only show positioning results with an interval of 20 steps. From the Figure 10, we can see that the overall errors of APFiLoc and PDR + Landmarks are decreasing with time. This is because the number of organic landmarks increases over time until all the organic landmarks in the space of interest are recognized. Initially, the errors of APFiLoc and PDR + Landmarks are large due mainly to lack of enough landmarks or the locations of organic landmarks are inaccurate. With more traces available, the locations of organic landmarks are refined and more organic landmarks are discovered, leading to an increase in the accuracy of both APFiLoc and PDR + Landmarks. Also, landmarks have less influence on the performance of APFiLoc than that on the PDR+Landmarks method since APFiLoc makes use of not only landmarks, but also map information. However, the performance of PF + Map + PDR method generally stays stable over time since it does not use the landmarks to refine the result.

## 8. Conclusions and Future Work

This paper presents an indoor positioning solution called APFiLoc that uses the APF to fuse readings of smartphone inertial sensors, map constraint, and landmarks. The LS-SVM is used to classify seed landmarks and a clustering algorithm relying on distance constraint is developed to recognize organic landmarks. This algorithm does not need the knowledge of the number of clusters. Our solution bypasses the troublesome effort for fingerprint collection and can achieve 80% accuracy (phone in the hand) and around 70% accuracy (phone in the pocket) of the error less than 2 m without Wi-Fi infrastructure requirement.

In contrast to prior studies about indoor positioning, which focus commonly on building a precise wireless signal propagation model or reducing the workload of collecting Wi-Fi fingerprints, our APFiLoc does not depend on Wi-Fi APs. This means that APFiLoc is suitable to cases in which it is impossible or impractical to install large amounts of fixed infrastructure into the environment in advance. This is because of the utilization of map information and landmarks. The map information allows APFiLoc to eliminate invalid particles, thereby improving the accuracy of positioning. Meanwhile, when a user passes through a landmark, his position can be calibrated and the parameter of Weinberg model is updated at the same time.

Despite the fact that our work can provide a relatively high accuracy, it requires the knowledge of maps. Therefore, in the future, we will use the crowdsourcing method to generate the spatial model for indoor localization that contains not only environmental constraints represented by a map, but also landmarks and so on. Besides, multiple neighbouring landmarks will be utilized in the phase of landmark matching, which would further reduce the location error. Another challenge that still waits to be solved is how to obtain accurate heading estimation, which is independent of different complex phone poses (e.g., phone in the bag, users swing the phone when moving) and motion states.
